# Telomere‐to‐telomere genome assembly of the endangered Jacktree (*Sinojackia xylocarpa* Hu) reveals its centromeric landscape and evolutionary dilemma as a small population

**DOI:** 10.1111/tpj.71186

**Published:** 2026-11-04

**Authors:** Si‐Di Liang, Si‐Si Zheng, Lei Wu, Ang Liu, Hong‐Hu Meng, Yun Jiang, Zheng‐Wei Wang, Bin‐Jie Ge, Wen‐Li Zou, Qian Wan, Xi‐Ling Dai, Gregor Kozlowski, Nai‐Wei Li, Xiao‐Ling Yan, Yi‐Gang Song

**Affiliations:** ^1^ Key Laboratory of National Forestry and Grassland Administration on East China Plant Conservation and Utilization Shanghai Chenshan Botanical Garden Shanghai 201602 China; ^2^ College of Life Sciences Shanghai Normal University Shanghai 200234 China; ^3^ College of Forestry Central South University of Forestry and Technology Changsha 410004 China; ^4^ Applied Research Center for Tropical Plant Conservation & Southeast Asia Biodiversity Research Institute, Xishuangbanna Tropical Botanical Garden Chinese Academy of Sciences Mengla 656303 China; ^5^ Administration Bureau of Longgan Lake National Nature Reserve Huangmei 435500 Hubei Province China; ^6^ Department of Biology and Botanic Garden University of Fribourg Fribourg Switzerland; ^7^ Natural History Museum Fribourg Fribourg Switzerland; ^8^ Institute of Botany Jiangsu Province and Chinese Academy of Sciences (Nanjing Botanical Garden Mem. Sun Yat‐Sen) Nanjing 210014 China

**Keywords:** *Sinojackia xylocarpa*, endangered plants, T2T, centromere, genomic erosion, genetic load, conservation biology

## Abstract

The endangered status of many plant species is often attributed to a decline in fitness, which drives a sustained decrease in population size under natural conditions. However, the role of highly repetitive genomic regions in the decline of endangered plants remains poorly understood. Here, we present the gap‐free telomere‐to‐telomere (T2T) reference genome for the endangered Jacktree (*Sinojackia xylocarpa* Hu). It has a genome size of 961.94 Mb, an N50 of 78.05 Mb, and 36 446 predicted protein‐coding genes. Using CENH3 chromatin immunoprecipitation sequencing (ChIP‐seq), we identified centromeric regions across all 12 chromosomes and revealed that *S. xylocarpa* centromeres are composed of diverse transposable element (TE) families, with long terminal repeat (LTR) retrotransposons and LINE elements as major components. Notably, the right arm of chromosome 1 exhibited ectopic CENH3 enrichment and a TE accumulation similar to that of centromeric regions, suggesting the formation of a centromere‐like chromatin domain. In addition, we detected inversion polymorphisms and inversion heterozygotes in natural populations, predominantly concentrated on the same chromosomal arm. These features may be associated with meiotic abnormalities. Using a high‐quality T2T genome assembly, we identified a pattern of genomic erosion facing these small populations characterized by local suppression of recombination linked LTR proliferation and by the accumulation of deleterious mutations in DNA repair genes. This genomic erosion creates an evolutionary dilemma that impairs the purging of deleterious alleles, ultimately threatening long‐term adaptability and survival. This study highlights the utility of T2T genome assembly for the conservation of endangered plants.

## Significance Statement

We report a T2T genome of endangered *Sinojackia xylocarpa*, whose centromeres comprise diverse transposable element (TE) families, and identify a centromere‐like domain and inversion polymorphisms on the chr1 right arm. Small population genomic erosion weakens purifying selection and creates an evolutionary dilemma, underscoring the T2T genome's value for endangered plant conservation.

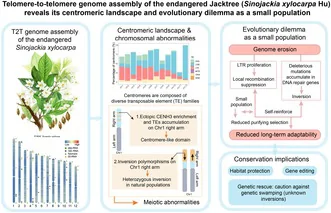

Our T2T genome of *Sinojackia xylocarpa* reveals centromeres composed of diverse TEs, with LTR retrotransposons and LINEs as major components. Recombination suppression linked to LTR accumulation and inversions has led to the accumulation of deleterious mutations in populations, particularly in DNA repair genes. This genomic erosion creates an evolutionary dilemma by preventing the purging of deleterious alleles, compromising the long‐term adaptability of small populations.

## BACKGROUND

A small population size is not merely a numerical descriptor but reflects a vulnerable state in which a population becomes susceptible to stochastic events and a negative feedback loop (Caughley, 1994). This vulnerability arises because small populations are subject to genetic drift, which leads to rapid loss of genetic diversity accompanied by inbreeding depression and accumulation of deleterious mutations (Dussex et al., 2023). The convergence of a range of extrinsic and intrinsic processes can form an “extinction vortex” (Bosse & van Loon, 2022; Fagan & Holmes, 2006). Population decline promotes inbreeding depression, causing further accumulation of deleterious mutations. This, in turn, reduces reproductive success and survival rates, making the population increasingly vulnerable to stochastic events, eventually driving the population to extinction. Owing to their narrow habitats and limited capacity to track shifting climate envelopes, plant species with small populations are expected to face a higher risk of climate‐driven extinction (Fricke et al., 2022; Jose, 2024; Wang et al., 2026). Therefore, it is necessary to assess the likelihood of endangered plants becoming trapped in an extinction vortex and to formulate targeted conservation strategies accordingly (Höglund, 2009).

Genomic erosion encompasses a set of genetic threats to small populations, including loss of genetic diversity, increased genetic load, and accumulation of maladaptive variants, and constitutes a link in the negative feedback loop in which small populations may become trapped (Gargiulo et al., 2025; Robinson et al., 2023; van Oosterhout et al., 2026). In recent years, a growing number of studies have focused on the role of genome features in the long‐term viability of small populations and have often identified genomic erosion in such populations (Jackson et al., 2022; Zhang et al., 2024). These findings suggest that genomic tools contribute essential knowledge to management and recovery programs for endangered species (Robinson et al., 2023). However, the evolutionary effects of highly repetitive sequences on small populations remain poorly understood, owing to the high density of repetitive sequences in telomeres, centromeres, and their adjacent regions, which has historically made complete assembly in these areas challenging (Cleveland et al., 2003; Henikoff et al., 2001; Malik & Henikoff, 2009; Miga, 2015; Naish, 2024). Recent advances in telomere‐to‐telomere (T2T) assembly technologies have enabled the resolution of complete telomeres and centromeres (Li et al., 2021; Naish et al., 2021). The application of T2T genome assemblies may offer new opportunities to elucidate the genomic mechanisms underlying self‐reproductive barriers to self‐renewal in endangered plant species.

Jacktree (*Sinojackia xylocarpa* Hu) is a deciduous tree with beautiful flowers and fruits (Figure 1a,b) belonging to *Sinojackia* Hu of Styracaceae (Hu, 1928). It is a unique species found in the lower reaches of Yangtze River which has been listed as vulnerable (VU) by the International Union for Conservation of Nature and Natural Resources (IUCN) Red List (https://www.iucnredlist.org/) (Zhu et al., 2024). The distribution of *S. xylocarpa* is limited and fragmented, and its populations are uniformly small (Wu et al., 2023). Previous studies have attributed its endangered status to low reproductive capacity, including prolonged seed dormancy (Wu et al., 2023; Zhu et al., 2024) and meiotic abnormalities (Wang et al., 2003). However, the genetic causes underlying the decline of Jacktree and the genomic risks faced by its current small populations remain unexplored. To address this gap, we assembled a T2T genome of *S. xylocarpa* to investigate the genomic drivers underlying its small population size. Additionally, population‐level analyses based on a gap‐free genome provide deeper insights into genomic erosion in endangered small populations.

**Figure 1:**
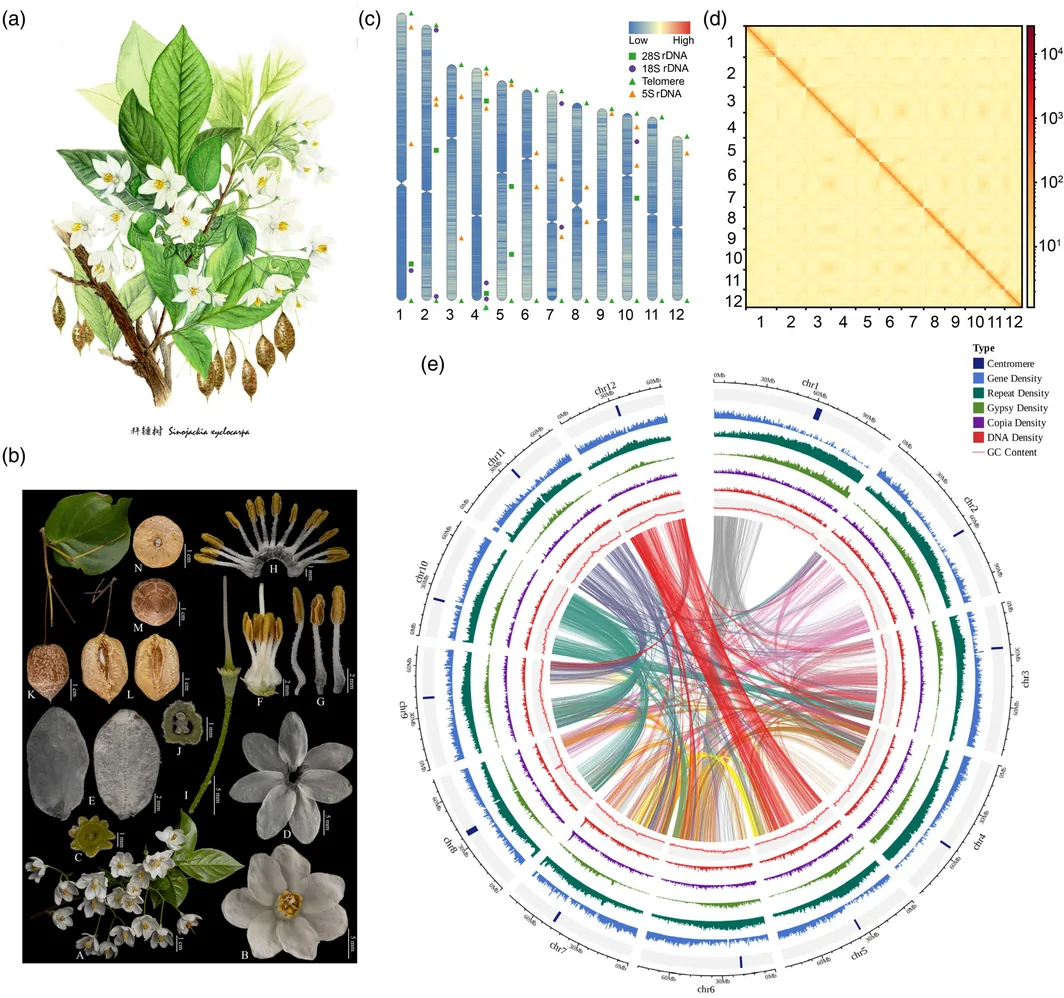
The assembly of T2T reference genome for *S. xylocarpa*. (a–b) Images of *S. xylocarpa* covered with white blooms and its fruits like a traditional steelyard balance of China. (c) Gene density (Low–High) and the distribution of telomeres, centromeres, 28S rDNA, 18S rDNA, and 5S rDNA in the *S. xylocarpa* genome. (d) The Hi–C interaction matrix of the assembly. (e) Genomic features of the *S. xylocarpa* genome. From the outside in, these are the centromere position, gene density, repeat density, Gypsy density, Copia density, DNA density, GC content, and distribution of genomic collinearity loci (homologous block in genome sequence).

In this study, we integrated PacBio HiFi reads, Oxford Nanopore Technologies (ONT) ultralong reads, and Hi‐C reads to complete the gapless genome of the endangered *S. xylocarpa*. To the best of our knowledge, this is the first publicly available T2T and gap‐free reference genome for the family Styracaceae. The T2T genome assembly revealed the fine‐scale features of highly repetitive regions, such as telomeres and centromeres, in an endangered plant species, laying a valuable foundation for understanding the overall genomic structure in threatened plants more broadly. By leveraging the gapless genome, we conducted high‐resolution population genomic analyses of *S. xylocarpa*, enabling the precise quantification of genomic variation and reconstruction of its demographic history, with particular resolution in previously inaccessible centromeric regions.

## RESULTS

### Assembly of *S. xylocarpa* diploid T2T genome

A high‐quality T2T genome assembly of *S. xylocarpa* was achieved by integrating multiple sequencing platforms. A total of 82.53 Gb (85.8× coverage) of high‐fidelity (HiFi) reads were generated using the PacBio Sequel II platform, with an average read N50 of 18.09 kb. In addition, ultralong reads were obtained using ONT, totaling 44.96 Gb (46.3× coverage) with a read N50 of 42.33 kb. To further support chromosome‐scale scaffolding, 93.40 Gb Hi‐C clean reads were generated, yielding over 200 million valid interaction contacts (Table S1).

Next, different assembly strategies were used for the genome assembly. We initially assembled the ONT ultralong data, whereas the HiFi data were assembled using two strategies: a pure HiFi assembly and mixed HiFi + ONT ultralong assembly. After a comprehensive evaluation, the contig‐level genome with the highest quality (Benchmarking Universal Single‐Copy Orthologs [BUSCO]  = 97.30%) using the HiFi‐only assembly strategy was selected as the T2T genome scaffold (Table S2). Based on the HiFi reads, we constructed a phased diploid assembly using HiFiasm, resulting in a contig size of 1 042 120 927 bp with an N50 of 78 050 245 bp. Using 93.4 Gb of Hi‐C reads, the assembled HiFi contigs were further grouped, ordered, and oriented into 12 chromosomes with a total length of 962 044 720 bp. After telomere extension, gap filling, and error correction, we obtained a gap‐free T2T reference genome totaling 961 939 874 bp (Table 1; Table S3; Figure 1c,d,e).

**Table 1:** Statistics of *Sinojackia xylocarpa* genome assembly

Genomic feature	*Sinojackia xylocarpa*
Total length (bp)	961 939 874
Number of contigs (gaps)	0
Total number	12
GC content (%)	38.86
N50 (bp)	78 050 245
Genome BUSCO (%)	97.30%
Total size of TE proteins (bp)	92 946 569
Number of predicted genes	36 446
Gene BUSCOs (%)	99.01%

### Genome annotation

We predicted 36 446 coding genes in the genome, with an average mRNA length of 7774.39 bp and an average CDS length of 1157.78 bp. Each gene had an average of 5.22 exons, with an average exon length of 297.24 bp, whereas the average intron length was 1470.41 bp (Table S4). The distribution of gene lengths in this species was similar to that observed in other Ericales species, indicating that our annotation results are reliable (Figure S1). Genome annotation captured 99.01% (1598 of 1614 BUSCOs) of the core conserved genes (Table 1; Table S5). We predicted gene functions, including motifs, domains, protein functions, and metabolic pathways, using publicly available functional annotation databases. Overall, the results indicated that 96.24% (35 076) of the genes were annotated in at least one database (Table S6).

In addition, we identified 201 microRNAs (miRNAs), 929 transfer RNAs (tRNAs), 1356 small nuclear RNAs (snRNAs), and 3139 ribosomal RNAs (rRNAs), which together accounted for 0.07% of the *S. xylocarpa* genome (Table S7). The tandem repeats (TRs) were 64 493 885 bp long and accounted for only 6.71% of the genome (Table S8). Transposable elements (TEs) spanned 65.97% of the genome, comprising 22.62% Gypsy, 8.20% Copia LTR retrotransposons, along with 0.82% short interspersed nuclear elements (SINEs; Table S8).

### Detection of highly repetitive sequences in chromosomes

Using 5‐Mb sliding windows to calculate density along chromosomes, we observed that highly repetitive sequences in *S. xylocarpa* are localized to specific chromosome regions (Figure S2). Regions with repeat density exceeding 90% were found on the first five chromosomes but not on the other seven. On chr1, repeat density surpassed 80% beyond 35 Mb, with an average of 94.30% observed on the right arm; on chr4, density was above 80% between 45 Mb and 85 Mb.

Telomeres were identified in all chromosomes through conserved repeats (5′‐CCCTAAA‐3′/5′‐TTTAGGG‐3′), with copy numbers ranging from 475 to 2089 (mean = 1083). There was substantial variation in telomeric repeat copy number among chromosomes (Table S9).

To identify the CENH3 homolog in *S. xylocarpa*, the Arabidopsis CENH3 (HTR12) protein sequence was used as a query to search against the predicted protein database. Among the candidate homologs, the transcript encoded by *SxylChr5G122060* exhibited the highest similarity to HTR12, with 71.88% amino acid sequence similarity. To validate its centromeric localization, we generated a polyclonal antibody against the N‐terminal region (2–18 amino acids) of the predicted CENH3 protein. Immunofluorescence (IF) showed distinct punctate signals associated with interphase nuclei, consistent with the expected localization pattern of CENH3 variants (Figure S3). These results confirmed that *SxylChr5G122060* encodes the functional CENH3 homolog in *S. xylocarpa* (hereafter referred to as SxyCENH3). To identify centromeric regions, we performed SxyCENH3 ChIP‐seq, and SxyCENH3‐enriched regions were defined based on genome‐wide enrichment profiles. Compared with chromosome arms, all identified centromeric regions exhibited SxyCENH3 enrichment with broad peaks, whereas surrounding regions showed substantially lower signals (Figure S4). The lengths of centromeres ranged from 0.94 Mb (chr5) to 3.01 Mb (chr1), representing 1.15% (chr5) to 4.07% (chr8) of the corresponding chromosome lengths (Table S9). We also identified 161 predicted protein‐coding genes in the centromeric region, with 68 on chr11 and none on chr6 (Table S9).

### Comparative genomics of *S. xylocarpa* within Ericales

Comparative genomic analysis across 16 species, including 13 from Ericales, identified 92 380 orthologous gene families comprising 612 438 genes (Figure 2a; Table S10). Among these, 84 were single‐copy gene families, and 6461 were conserved across all 16 species, totaling 233 617 genes. *S. xylocarpa* contained 3205 unique gene families (4459 genes), with GO enrichment indicating functions related to acid phosphatase activity, and KEGG analysis highlighting pathways, including carbon metabolism and glycolysis/gluconeogenesis (Figure S5A). In comparison with *A. chinensis*, *C. sinensis*, and *R. vialii*, unique gene families in *S. xylocarpa* were enriched in the Elongator holoenzyme complex and were associated with the carbon metabolism pathway (Figure 2c; Figure S5B).

**Figure 2:**
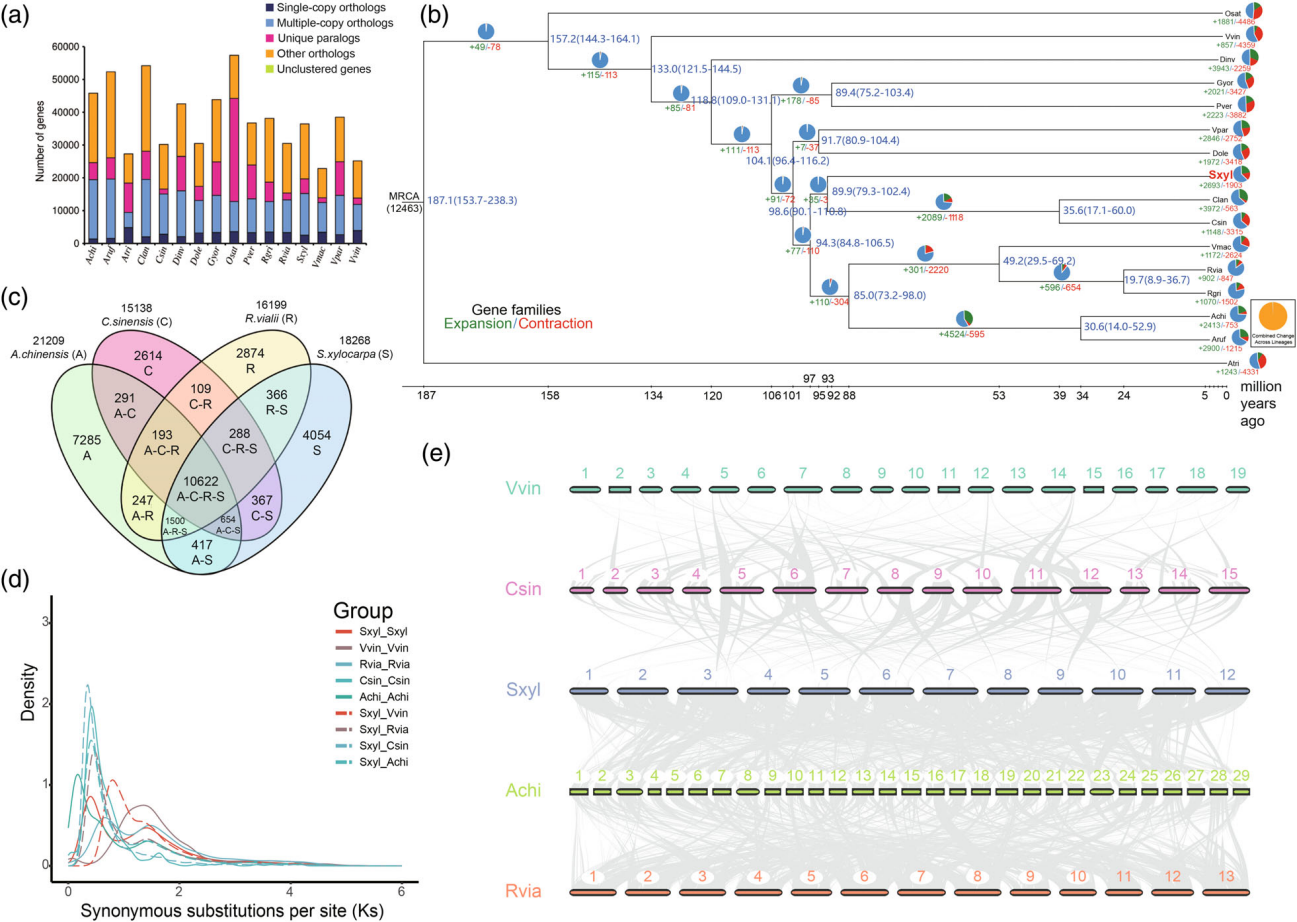
Comparative genome and evolution analysis of 16 species. (a) Gene family clustering. (b) The phylogenetic tree, divergence times, and gene family contraction and expansion of all 16 species. (c) Venn diagram of shared and unique gene families of four species. (d) WGD events detection inferred from synonymous substitutions per synonymous site (*K*_s_). (e) Gene‐based genome collinear comparison among *V. vinifera*, *C. sinensis*, *S. xylocarpa*, *A. chinensis*, and *R. vialii*.

Gene family expansion in *S. xylocarpa* involved 2693 families (10 382 genes), enriched in UDP‐glycosyltransferase activity, pollen recognition, and carbohydrate binding, and associated with pathways including zeatin biosynthesis, glutathione metabolism, and stilbenoid, diarylheptanoid, and gingerol biosynthesis (Figure S5C). Conversely, 1903 gene families (2011 genes) were contracted (Figure 2b). The enrichment of unique and expanded gene families suggests that *S. xylocarpa* has undergone significant diversification in its signal transduction processes, particularly those involving UDP‐glycosyltransferase activity and pollen recognition.

### Genome evolution of *S. xylocarpa*

A maximum likelihood (ML) phylogenetic tree was constructed based on 127 strict single‐copy orthologous genes from 16 species (including 13 representatives from Ericales) (Figure 2b). The analysis revealed that Styracaceae was most closely related phylogenetically to Theaceae, with an estimated divergence time of approximately 89.9 million years ago (Ma). This was preceded by the divergence between Ericaceae and Actinidiaceae around 94.3 Ma, suggesting the rapid radiation of major Ericales lineages during the Late Cretaceous (Figure 2b). Whole‐genome duplication (WGD) analyses inferred two rounds of WGD in *S. xylocarpa*: one ancient event shared by all examined species and another more recent event shared with *C. sinensis* (Figure 2d). In contrast, *A. chinensis* experienced three WGD events, which is consistent with its higher chromosome number (*n* = 29) than that of other species. These findings suggest that the earliest WGD event occurred in the common ancestor of the core eudicots, whereas a subsequent duplication was shared by both Theaceae and Styracaceae. Our findings do not support the hypothesis that Styracaceae species with *n* = 12 originated from the recent polyploidization of *Styrax* (*n* = 8; Deng, 2024). Instead, they support the notion that independent diversification events occur among genera within the family. Genomic collinearity analysis further supported this relationship, with stronger collinearity observed between *S. xylocarpa* and *C. sinensis* than between *S. xylocarpa* and *A. chinensis* (Figure 2e). Finally, *SxylChr2G042980* (4‐alpha‐glucanotransferase) and *SxylChr11G332510* (non‐specific phospholipase C2) were identified as being under significant positive selection and were potentially involved in glycerolipid metabolism, starch and sucrose metabolism, and hydrolase activity on ester bonds, suggesting adaptive evolutionary responses in these functional pathways (Figure S5E; Table S11).

### Landscape of centromeres

Our results showed that centromeric regions in *S. xylocarpa* were predominantly composed of TEs, accounting for 73.73% of the centromeric sequences (Figure 3a,b). Among these, LINEs and LTR retrotransposons were the most abundant, representing 31.58% and 23.58% of centromeric TE content, respectively (Figure 3b). SxyCENH3 enrichment in TE regions was significantly higher than that in non‐TE regions (Figure 3c,d). Within LTR retrotransposons, the Gypsy superfamily (17.78%) was predominant, with the Ogre and Tekay subfamilies being widely distributed across all centromeres (Figure S6). Although LINE copies (8527) outnumbered LTR copies (5181), LTR retrotransposons exhibited stronger SxyCENH3 enrichment signals, suggesting that LTR retrotransposons were likely the primary contributors to CENH3 binding (Figure 3e,f). Notably, all centromeric TE superfamilies showed significant SxyCENH3 enrichment (Figure 3e), suggesting that multiple TE families may collectively contribute to centromere structural maintenance in this species.

Interestingly, we observed an asymmetric SxyCENH3 enrichment pattern between the left and right arms of chr1: the left arm exhibited near‐background enrichment levels (mean = −0.068), whereas the right arm showed significantly elevated enrichment (mean = 0.478), which was substantially higher than the genome‐wide average of non‐centromeric regions (−0.148; Figure 3g). Further analysis revealed that 16.39% of the right arm consisted of highly enriched regions (log_2_(ChIP/Input) >1), of which 98.39% of the total length was located within TEs. Among these, 82.42% of this length corresponded to unclassified transposons (6217 copies) and 12.66% to LTR/Gypsy (5866 copies; Figure S7). This TE composition, which was highly similar to that of bona fide centromeric regions, gave the right arm a centromere‐like character, potentially reflecting ectopic SxyCENH3 mislocalization outside the true centromeres.

**Figure 3:**
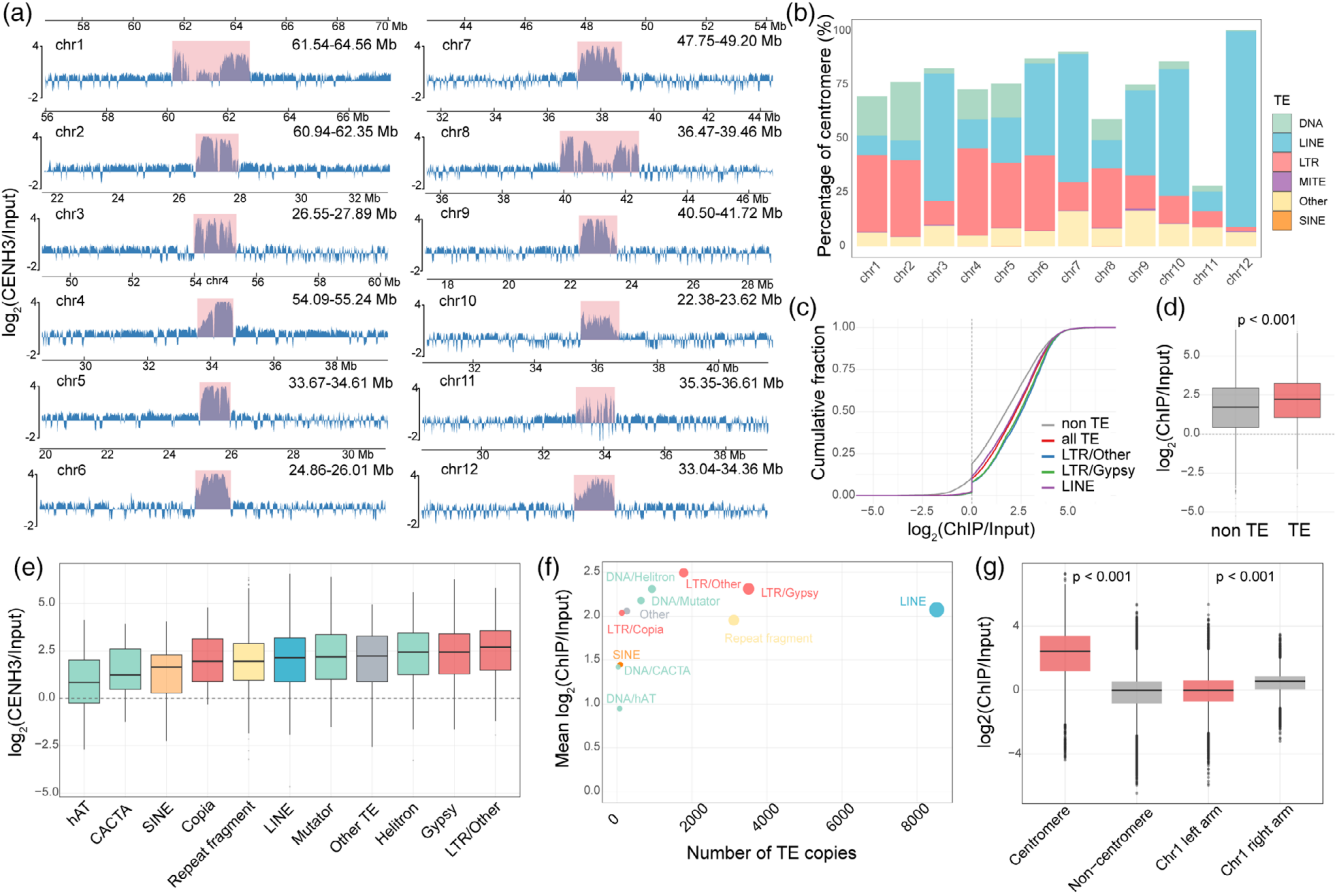
The landscape of centromeres. (a) SxyCENH3 ChIP‐seq enrichment profiles (±5 Mb flanking regions of the predicted centromeres). Enrichment values are shown as log_2_(ChIP/Input). The predicted centromeric regions are highlighted in pink. (b) Composition of transposable element (TE) superfamilies within each chromosome's centromeric region, shown as the proportion of total TE length. (c) Cumulative distribution of log_2_(ChIP/Input) enrichment values for non‐TE regions, all TEs, LTR/Other, LTR/Gypsy, and LINE elements within the centromeric regions. (d) Boxplot comparing log_2_(ChIP/Input) enrichment between all TE and non‐TE regions within centromeres. (e) Boxplot showing log_2_(ChIP/Input) enrichment across different TE superfamilies identified by EDTA2 annotation. (f) Scatter plot depicting the relationship between mean log_2_(ChIP/Input) enrichment and the number of TE copies for each TE superfamily. (g) Boxplot comparing log_2_(ChIP/Input) enrichment among centromeric regions, non‐centromeric regions, and the left and right arms of chromosome 1. All statistical comparisons were performed using the two‐sided Wilcoxon rank‐sum test.

### Population genetics of *S. xylocarpa*

Comprehensive whole‐genome sequencing was performed on 32 individuals from eight known populations of *S. xylocarpa* (Table S12; Figure 4a). We identified a total of 67.34 Mb of high‐confidence variants, including 62 502 573 single‐nucleotide polymorphisms (SNPs), 1 809 744 insertions, and 3 030 722 deletions (Table S13). Among these, transversion predominated (56.90%; Figure S8B), whereas the distribution of indel lengths exhibited a bimodal pattern with peaks at 1–4 and 10 bp (Figure S8C). In addition, the genome‐wide distribution of genetic variation showed significant heterogeneity. We observed a sharp decline in polymorphism within specific genomic regions, including the distal part of chr1, the middle part of chr2, and the distal part of chr4 (Figure S9). Functional annotation classified the variants into 15 618 498 intergenic, 3 705 735 intronic, and 484 332 exonic categories (Table S14; Figure S8A), with coding variants including 277 673 non‐synonymous and 189 300 synonymous mutations (non‐synonymous/synonymous ratio = 1.467).

**Figure 4:**
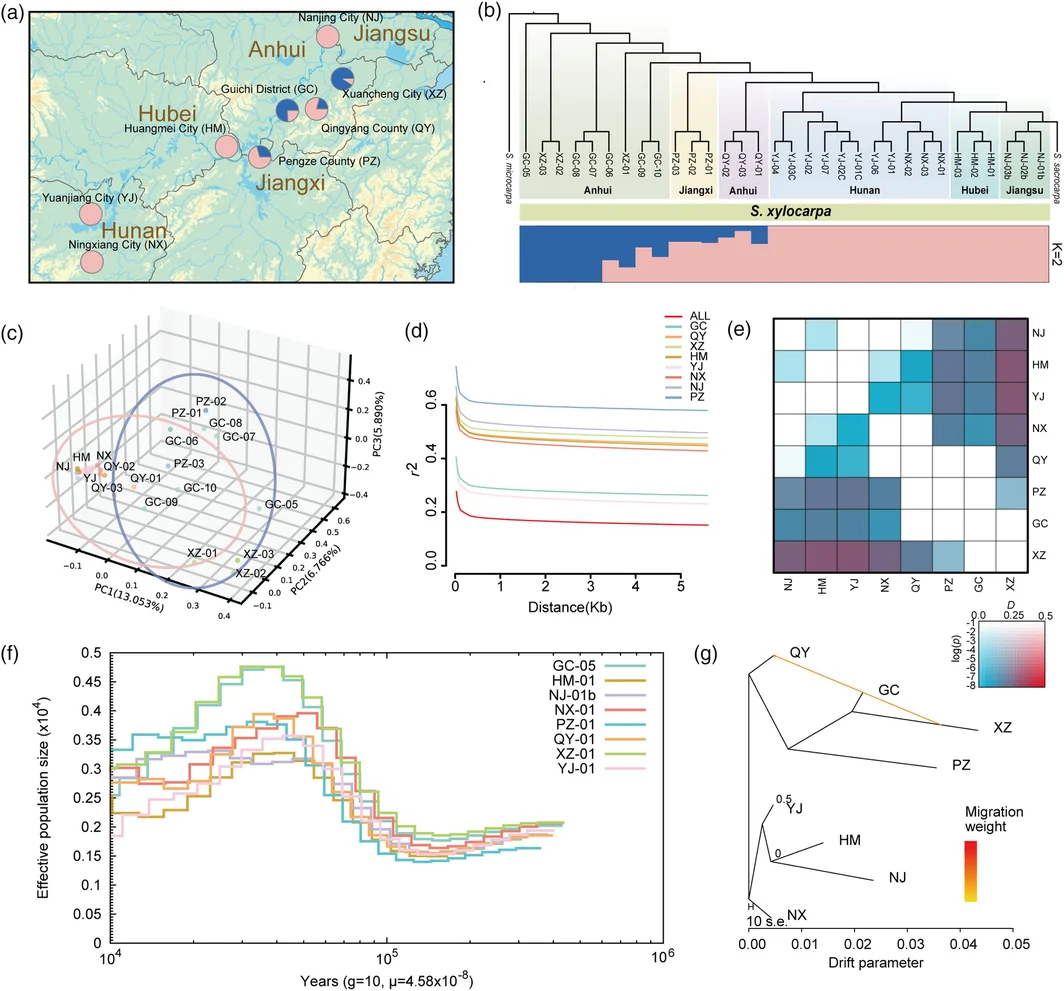
Population genetic analysis of *S. xylocarpa*. (a) The distribution of the eight populations and the average genetic structure of *S. xylocarpa* inferred using ADMIXTURE. (b) The phylogenetic tree of 32 samples of *S. xylocarpa*, *S. sarcocarpa*, and *S. microcarpa* (outgroup). The clades were named by the province of distribution of populations corresponding to the map on the left. The genetic structure of each sample at the bottom was set at *K* = 2. (c) Principal component analysis (PCA) of all samples. (d) Linkage disequilibrium (LD) decay at 5 kb length. (e) Assessment of gene flow between populations using the *D* statistic. (f) The population size history of *S. xylocarpa*. (g) Gene flow prediction between populations using TreeMix.

Phylogenetic reconstruction based on ML identified four geographically distinct clades of *S. xylocarpa*: Nanjing‐Jiangsu, Hubei, Hunan, and Jiangxi‐Anhui regions of China (Figure 4b). Principal component analysis (PCA) and ADMIXTURE identified two genetic clusters and a mixed genetic cluster (Figure 4b,c; Figure S10). Population genomic analyses showed rapid linkage disequilibrium (LD) decay (*r* ^2^ = 0.1492 at 5 kb) across most populations (Figure 4d; Figure S11). Remarkably, the PZ population exhibited exceptional LD persistence (Figure 4d). Genome‐wide *F*_ST_ and *π* analyses demonstrated strong selection signatures on chr1 and chr4 in the NJ‐GC/XZ comparison (Figure S12). Migration analysis revealed gene flow between the two genetic clusters identified by ADMIXTURE, as well as between QY and XZ (Figure 4e,g; Figure S13). The frequency of runs of homozygosity (*F*_ROH_) across samples ranged from 0.0091 to 0.0281, with the NJ and HM populations showing the highest mean *F*_ROH_ (0.0196 and 0.0221, respectively; Table S15; Figure S14). Concurrently, these two populations exhibited the lowest nucleotide diversity (*π* = 0.0032 and 0.0035, respectively), consistent with higher levels of inbreeding accompanied by reduced genetic diversity (Table S16). Demographic history revealed a peak effective population size (*N*_e_) of approximately 20 ka (Last Glacial Maximum), followed by a continuous decline, with most populations showing recovery after approximately 10 ka (Figure 4f).

### Chromosomal inversion in *S. xylocarpa* population

By comparing the previously published genome of *S. xylocarpa* with the T2T genome assembled in this study, we observed chromosomal inversions on most chromosomes, particularly in the distal region of chr1 (Figure S15). Notably, an inversion was detected in the upstream telomeric region of chr2. To further investigate inversion patterns within *S. xylocarpa* populations, we divided chr1 into left and right arms (Figure 5). The mean observed heterozygosity (*H*_o_) was 0.25 for the left arm and 0.20 for the right arm (Table S17; Figure 5a). Using PCA, we found that the population structure based on the left arm was similar to that of the whole‐genome PCA results (Figure 5b). However, for the right arm, the *S. xylocarpa* population was divided into three clusters, with the middle cluster comprising individuals GC‐06, GC‐10, QY‐01, and XZ‐01 (Figure 5b). These four individuals all had *H*_o_ values >0.5 in the right arm (Figure 5a). These results suggest the presence of heterozygous inversion individuals in the GC, QY, and XZ populations.

**Figure 5:**
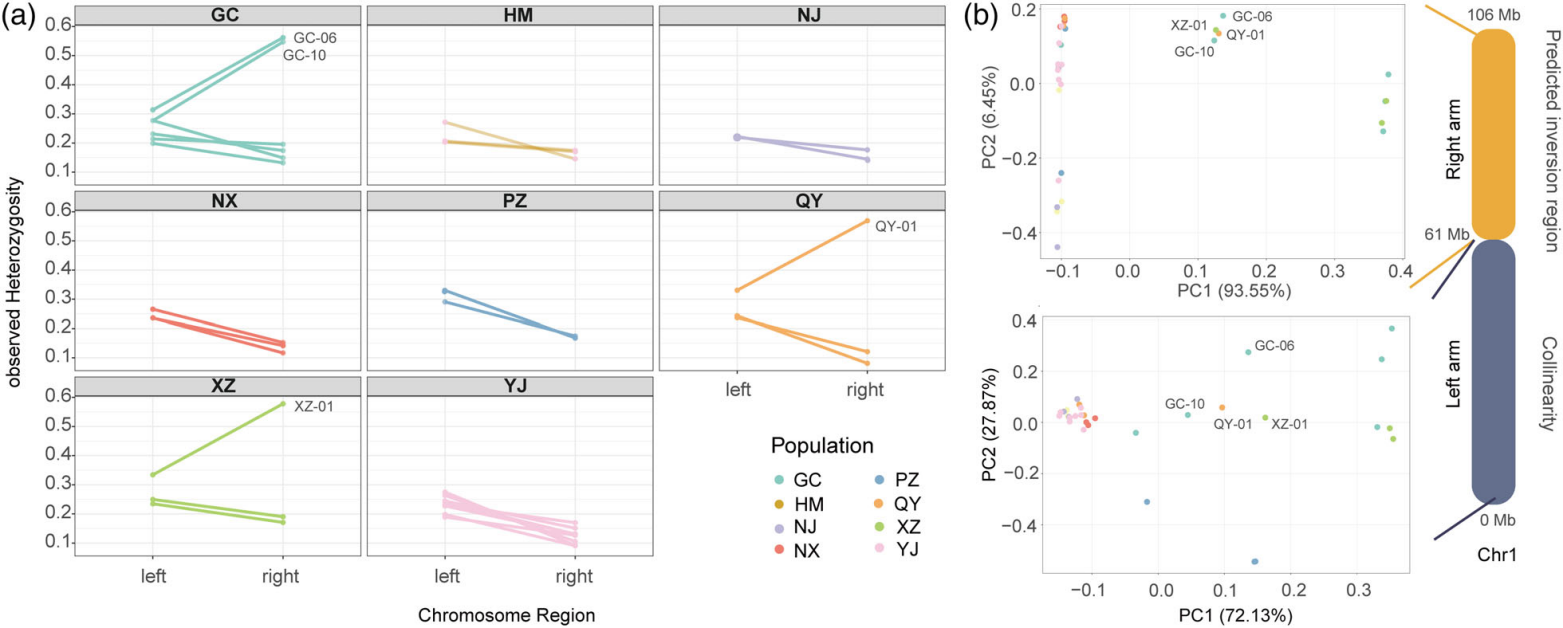
Chromosomal inversion in *S. xylocarpa* population. (a) Comparison of *H*_o_ between the left arm and right arm of chr1 across 32 individuals. (b) PCA of the putative inversion region (right arm) and the collinear region (left arm) of chr1, revealing distinct genetic structure between populations. Individuals are colored according to their population.

### Genomic recombination landscape and deleterious mutation accumulation in *S. xylocarpa*

Using genome‐wide SNP data from all *S. xylocarpa* populations, we estimated recombination rates (*ρ*) in non‐overlapping 100‐kb windows to reconstruct the genome‐wide recombination landscape. Genome‐wide recombination rate analysis revealed a 10th percentile threshold of *ρ* = 180.14 (Table S18; Figure S16). Two peaks were observed at *ρ *= 196.57 (with the ±20 interval covering 24.2% of the genome) and *ρ* = 386.96 (with the ±20 interval covering 14.1%), indicating that a large proportion of the genome falls outside these high‐recombination regions and likely experiences suppressed recombination. The analysis revealed significant heterogeneity in the recombination rates across the genome, with extensive chromosomal regions exhibiting markedly inhibited recombination (Figure 6a).

**Figure 6:**
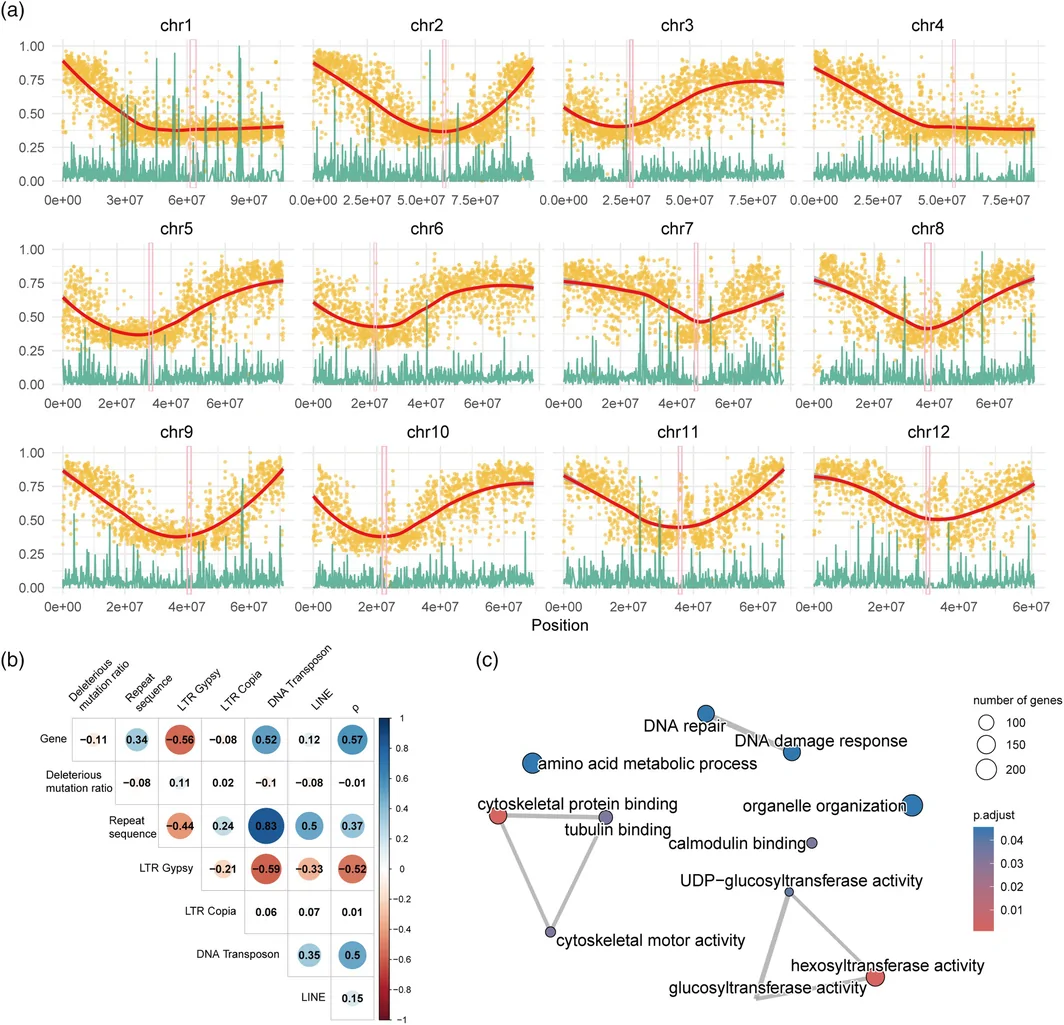
Genomic recombination landscape and deleterious mutations. (a) The genomic landscape illustrates recombination rates (depicted by yellow dots) and the deleterious mutation ratio (shown as a green line) across non‐overlapping 100‐kbp windows. All data were subjected to normalization procedures. The pink box marks the position of the centromeres, while red lines represent loess‐smoothed recombination rates. (b) A Pearson correlation coefficient matrix among deleterious mutation ratio, recombination rates (*ρ*), and the density of genomic features. (c) The GO enrichment analysis of genes harboring deleterious mutations.

We also predicted deleterious mutations using SIFT4G and identified 68 655 such mutations. Although no significant genome‐wide correlation was detected between the recombination rate and deleterious mutation ratio (*r* = −0.01, *P* = 0.487), localized enrichment of deleterious mutations was observed in certain low‐recombination regions, particularly in the mid‐to‐distal portion of chr1 (Figure 6a).

To investigate the potential genomic determinants of these patterns, we constructed a correlation matrix incorporating various genomic features, recombination rates, and deleterious mutation rate densities (Figure 6b). The recombination rate was positively correlated with the gene count (*r* = 0.57, *P* < 1e^−16^), whereas Gypsy‐type LTR elements were negatively correlated with recombination (*r* = −0.52, *P* < 1e^−16^). In contrast, the ratio of deleterious mutations showed virtually no correlation with other genomic features (Figure 6b).

GO enrichment analysis of genes harboring deleterious mutations revealed functional clustering: 125 genes were involved in DNA repair, 190 in amino acid metabolic processes, and 130 in DNA damage response pathways (Figure 6c; Table S19). Among them, a deleterious mutation was identified in *POLH* (*SxylChr1G021390*), located in the central region of chr1; *POLH* is involved in the repair of ultraviolet‐induced DNA damage (Santiago et al., 2008; Bi et al., 2021; Figure S17). No homozygous individuals carrying this mutation were detected within the population, suggesting a lethal phenotype or reduced fitness (Figure S18). Additionally, in the upstream telomeric inversion region of chr2, deleterious mutations were identified in four DNA repair genes, including *RAD51* (*SxylChr2G024090*), *RFA1* (*SxylChr2G025250*), *RecA* (*SxylChr2G025780*), and *RAD7* (*SxylChr2G025090*).

## DISCUSSION

### Chromosomal abnormalities in *S. xylocarpa*

In the T2T genome of *S. xylocarpa*, high‐density repeats are concentrated on chr1, where a right arm region comprises 94.30% repetitive sequences (Figure 1e; Figure S2). The SxyCENH3 signal enrichment on the right arm of chr1 is significantly higher than the genome‐wide non‐centromere level, suggesting potential ectopic localization of CENH3 (Figure 3g; Figure S4). Comparative analysis of the two genomes of *S. xylocarpa* revealed large‐scale chromosomal inversions in the right arm of chr1 (Figure S15). Additionally, four individuals (GC‐06, GC‐10, QY‐01, and XZ‐01) exhibited high heterozygosity (>0.5) and were separated from other genetic clusters along PC1 in the right arm PCA of chr1, suggesting that these individuals are heterozygous for an inversion in this region (Figure 5). Genome‐wide *F*_ST_ and *π* analyses further demonstrated selection signatures in the right arm of chr1 in the NJ‐GC/XZ comparison (Figure S12).

Chromosomal abnormalities, such as chromatin bridges and fragments, occur during meiosis in *S. xylocarpa* (Wang et al., 2003). Comparative analysis of the two *S. xylocarpa* genomes revealed numerous chromosomal inversions, providing indirect genomic evidence consistent with the cytologically observed meiotic abnormalities, although the frequency of such events in natural populations remains unclear. Based on T2T genomic and population analyses, we propose several mechanisms that may contribute to meiotic chromosomal abnormalities in this species.

First, the right arm of chr1 exhibits a relatively high level of SxyCENH3 enrichment, and its highly enriched regions are predominantly composed of TEs, resembling the centromeric regions. This transposon‐rich sequence environment may provide a permissive substrate for the ectopic recruitment of CENH3 nucleosomes. Aberrant localization of CENH3 at non‐centromeric regions can induce the formation of neocentromeres, interfere with normal centromere function, and consequently lead to chromosome segregation errors and genomic instability (Sharma et al., 2019). For instance, it may promote the formation of dicentric chromosomes, which are prone to generate chromatin bridges and chromosome breakage during cell division (Cleal & Baird, 2020; Jiang & Chan, 2024; Murnane, 2012). Therefore, the centromere‐like structure on the right arm of chr1 may represent a potential source of chromosomal structural instability. Nevertheless, future cytological analyses (such as CENH3 immunolocalization, kinetochore protein detection, and chromosome behavior observation) are still required for further validation. Second, the existence of inversion heterozygotes within the population is likely a key cause of meiotic abnormalities. During pairing of inversion‐heterozygous chromosomes, the inverted region fails to align linearly with its homolog, forming an inversion loop. In paracentric inversion heterozygotes, single crossovers within the inversion loop typically generate recombinant chromatids that are dicentric and acentric, resulting in the formation of chromatin bridges and acentric fragments at anaphase (Huang & Rieseberg, 2020; McClintock, 1938). These aberrant chromosome structures may impair gamete formation and ultimately reduce individual fertility.

Chromosome inversions arise from DNA double‐strand breaks (DSBs) (Kirkpatrick, 2010; Steckenborn & Marques, 2025). In this study, the high‐density repetitive sequences on chr1 may act as endogenous sources of replication or recombination stress, inducing DSBs during crossing over or recombination (Farkash & Luning Prak, 2006; Kim & Mirkin, 2013; Liao et al., 2023). More critically, *S. xylocarpa* has accumulated deleterious mutations in genes involved in DNA repair and the replication stress response (Figure 6c). For example, deleterious mutations in *RAD51* may reduce the efficiency of homologous recombination repair (Li & Ma, 2006; Zhao et al., 2026). Such functional defects compromise high‐fidelity homologous recombination and may shift DSB repair toward the error‐prone non‐homologous end‐joining (NHEJ) pathway (Jiang et al., 2025; Jiang & Chan, 2024). This error‐prone repair, particularly when multiple break sites are present simultaneously, can directly lead to the rotation and re‐joining of broken fragments in the reverse orientation, ultimately generating chromosomal inversions. Therefore, high‐density repetitive sequences may serve as triggers of chromosomal abnormalities, whereas deficiencies in the DNA repair system constitute the primary cause of their occurrence.

Notably, chromosomal abnormalities are also drivers of genome evolution (Benítez‐Benítez et al., 2025; Castellano‐Pozo & Comaills, 2025). Mechanisms of chromosomal rearrangement can lead to chromosome fusions that generate new chromosomes, as well as structural variations, including inversions, translocations, and deletions (Benítez‐Benítez et al., 2025; Stimpson et al., 2012). The distinct population structure at the right arm of chr1 suggests the existence of different chromosomal inversion architectures within *S. xylocarpa*, consistent with the observation that this region is under selection between the NJ‐GC and XZ genetic clusters. This suggests that inversions arising from chromosomal instability may drive ecological differentiation (Kissmer & Gompert, 2025). Inversions may also rapidly fix beneficial allele combinations, thereby facilitating rapid environmental adaptation (Zhang et al., 2026). Furthermore, studies have shown that mutations in DNA repair genes can drive speciation through chromosome fusion (Wan et al., 2025). Therefore, it is plausible that inversions arising from DNA repair gene dysfunction contribute to adaptive evolution in *S. xylocarpa*.

Future studies are needed to elucidate the associations among ecological differentiation, chromosomal abnormalities, and mutations in DNA repair genes in *S. xylocarpa*. First, it is necessary to assess the frequency of chromosomal abnormalities in natural populations, identify their underlying causes, and evaluate their impact on population fitness. The T2T genome we have generated provides a valuable tool for designing telomere and centromere DNA probes to observe meiotic chromosomal behavior using fluorescence in situ hybridization (FISH). Second, employing population pan‐genomic and population genetic analyses will allow us to investigate whether inversion patterns within populations are associated with ecological adaptation. Such efforts will enable us to assess the long‐term effects of chromosomal instability on the adaptive potential of this lineage, within the broader framework of conservation and evolutionary biology.

### Self‐reinforcing evolutionary dilemma in a small population of *S. xylocarpa*

Jacktree grows along the Yangtze River basin (Figure 4a). Its fruits possess a lignified pericarp that enables water‐mediated dispersal (Zhu et al., 2024). Demographic history indicates that *S. xylocarpa* reached its peak population size during the Last Glacial Maximum (Figure 4f), suggesting that seed dormancy and the lignified pericarp may be adaptations to cold and arid environments (Wu et al., 2019). However, the species has since experienced a progressive decline in effective population size (Figure 4f). Given these observations, we investigated genomic erosion in *S. xylocarpa* to explore the potential genetic factors contributing to its vulnerability.

We identified a distinctive genomic architecture characterized by suppression of recombination across specific genome regions (Figure 6a). This phenomenon is particularly pronounced in a specific region of chr1, which shows markedly fewer syntenic segments relative to other chromosomes, low gene density, high repeat density, and significantly reduced population variation (Figure 1e; Figure S9). This region also showed a significantly elevated ratio of deleterious mutations (Figure 6a). Further analyses revealed that the recombination rate is significantly negatively correlated with Gypsy‐type LTR density (Figure 6b). This observation is consistent with previous reports that LTR accumulation can locally suppress recombination (Nambiar & Smith, 2016). In addition, the ratio of non‐synonymous to synonymous substitutions across the population was substantially higher than expected, consistent with an accumulation of deleterious mutations (Shen et al., 2022), particularly in genes involved in essential biological processes such as DNA repair and the DNA damage response (Figure 6c). Notably, the genes harboring these deleterious mutations are implicated in UV‐induced DNA damage repair, including *POLH*, *RFA1*, and *RAD7*, suggesting increased susceptibility to UV‐induced damage (Lahari et al., 2018; Santiago et al., 2008; Smith et al., 2000).

This genomic erosion in *S. xylocarpa*, characterized by suppressed recombination and the accumulation of deleterious mutations, likely results from multiple interacting factors. First, the high density of Gypsy‐type LTR retrotransposons in recombination‐suppressed regions suggests that TE expansion has played a primary role in shaping the genomic landscape. The proliferation of LTRs may directly suppress local recombination rates by promoting heterochromatin formation and altering chromatin architecture (Nambiar & Smith, 2016). Notably, the localized enrichment of CENH3 on the right arm of chr1 may provide evidence for this heterochromatin remodeling. Second, deleterious mutations are enriched in genes involved in DNA repair and the DNA damage response. This finding implies that chromosomal breaks arising during recombination, particularly in LTR‐rich regions, may not be efficiently repaired. Such repair failure can lead to chromosomal inversions. When gene flow occurs between populations carrying different inversion arrangements, the resulting inversion heterozygotes may experience meiotic abnormalities, which could lead to reduced fertility. This mechanism may represent an additional factor contributing to the limited gene flow observed among *S. xylocarpa* populations. Moreover, a compromised DNA repair system could further contribute to the accumulation of deleterious mutations. Consequently, mutations in these essential repair genes can significantly reduce individual fitness. For instance, mutations in *POLH* may be particularly detrimental under future environments with increased UV radiation. Third, from a life history perspective, the fruit traits of *S. xylocarpa* are adapted to cold and arid environments; however, this propagation mechanism results in prolonged seed dormancy, leading to small, geographically isolated populations (Wu et al., 2023; Zhang et al., 2012). Small population size reduces the efficacy of natural selection (Leroy et al., 2021), especially in long‐lived trees such as *S. xylocarpa* (Byers & Waller, 1999).

Together, these three factors, LTR‐driven recombination suppression, defective DNA repair leading to inversions and mutation accumulation, and small population effects that reduce purifying selection, reinforce each other, creating the vulnerability observed in *S. xylocarpa*. Recombination suppression hinders the purging of deleterious mutations (Burri, 2021) and limits the influx of beneficial alleles from other populations (Aguillon et al., 2022; Moran et al., 2021). These effects are further exacerbated by defective DNA repair mechanisms, which promote the accumulation of harmful mutations and facilitate chromosomal inversions. Furthermore, inversions may induce chromatin bridge formation, which in turn promotes the accumulation of repetitive sequences (Zhang et al., 2026). This feedback pattern is consistent with what we observed in chr1. We hypothesize that this recombination suppression may arise from crossover inhibition, serving as a mechanism to mitigate chromosomal rearrangements. Collectively, these observed patterns form a self‐reinforcing evolutionary dilemma: small population size reduces the efficacy of natural selection; strong regional recombination suppression and mutations in DNA repair genes lead to the accumulation of deleterious mutations. These mutations may further impair the repair of chromosomal breaks and DNA damage, which in turn leads to reduced fitness. This creates a vicious cycle that ultimately leads to reducing both individual fitness and long‐term population viability. This cycle links the unique life history traits, demographic history, and genomic architecture of *S. xylocarpa* to its current endangered status.

Whether disruptions in recombination repair mechanisms underlie the widespread recombination suppression observed in the *S. xylocarpa* genome remains to be determined. Further research is required to elucidate the causal relationships between DNA repair defects, LTR proliferation, recombination suppression, and the aforementioned chromosomal abnormalities, and to assess whether these genomic features are shared among other endangered species with similar demographic histories.

### Conservation implications revealed by the T2T genome assembly

Our findings suggest that certain genomic features and deleterious mutations in *S. xylocarpa* may have reduced its fitness. These genomic patterns and the observed vulnerability to current environmental conditions likely result from multiple interacting factors that collectively create a self‐reinforcing dilemma. Therefore, under the current trajectory of rapid climate change, including rising temperatures and ultraviolet radiation, *S. xylocarpa* may be pushed further toward an extinction vortex.

Urgent conservation action is needed to prevent extinction driven by the combined effects of these internal genomic factors and external environmental pressures, beginning with stricter protection against habitat disturbance and illegal logging to halt further population decline caused by anthropogenic pressures. Our results indicate that the NJ and HM populations, which exhibit the highest inbreeding coefficients and the lowest genetic diversity (Tables S15 and S16; Figure S14), should be prioritized for conservation.

Genetic rescue efforts are beneficial for introducing genetic diversity and purging deleterious mutations (Whiteley et al., 2015). A commonly applied genetic rescue management strategy involves translocating individuals from other populations into a small population (Robinson et al., 2023). However, in *S. xylocarpa*, the introduction of allopatric individuals must be approached with caution due to the possible presence of unknown inversions between populations. While introducing inversion‐carrying individuals may increase genetic diversity, it could also reduce fertility in an already vulnerable small population, potentially leading to genetic swamping (Pérez‐Pereira et al., 2022; Robinson et al., 2018; Schneider & Cannon, 2025; Waller, 2015). An alternative strategy for demographic rescue is to introduce individuals from the same population (Whiteley et al., 2015). This approach can increase the number of individuals subject to purifying selection, thereby accelerating natural population renewal. Moreover, as inbreeding intensifies in small populations, deleterious recessive alleles are expressed more frequently, which over time may facilitate inbreeding purging and reduce the genetic load (Hedrick & Garcia‐Dorado, 2016). Therefore, for *S. xylocarpa*, artificially breaking seed dormancy could be used to propagate individuals from within the same population for subsequent reintroduction into the wild.

Recently, gene editing technologies have emerged as promising tools for conserving endangered plants. A recent study involving 23 species demonstrated the feasibility of gene editing in conservation biology, highlighting its potential as a novel strategy for plant preservation (Yin et al., 2024). These technologies can be used to repair deleterious mutations in DNA repair genes themselves. Monitoring the outcomes of such gene editing interventions facilitates the conservation of threatened species and provides valuable insights into fundamental questions regarding genome stability and evolution. However, such interventions require careful ecological and ethical evaluation to avoid unintended risks.

## MATERIALS AND METHODS

### Plant sample collection and sequencing

Fresh, healthy, young leaves of *S. xylocarpa* were collected and individually packaged for PacBio HiFi, ONT Ultralong, and Hi‐C sequencing. Immediately after collection, the samples were flash‐frozen in liquid nitrogen and stored at −80°C until further processing.

High‐molecular‐weight genomic DNA (gDNA) was extracted from each sample using a cetyltrimethylammonium bromide (CTAB) protocol. For PacBio HiFi sequencing, an SMRTbell library was prepared using 50 μg of gDNA with the SMRTbell Express Template Prep Kit 2.0 (Pacific Biosciences, CA, USA) following the manufacturer's instructions. Sequencing was performed using the PacBio Sequel II platform. For ONT sequencing, large DNA fragments were enriched using the Short Read Eliminator XL Kit (Circulomics, MD, USA) according to the ONT Community protocol. Library preparation was conducted using the SQK‐LSK109 kit (Oxford Nanopore Technologies, UK), and sequencing was performed using a PromethION flowcell. A Hi‐C library was constructed and sequenced according to a previously established protocol (Lieberman‐Aiden et al., 2009).

For RNA‐seq, six tissues (roots, stems, leaves, tender buds, flowers, and fruits) were collected at different developmental stages from *S. xylocarpa* individuals cultivated in the Shanghai Chenshan Botanical Garden. Total RNA was extracted using an RNAprep Pure Plant Kit (TIANGEN, Beijing, China). The RNA‐seq library was prepared as described by Zhong et al. (2011).

### Genome assembly and gap filling

Preliminary contig‐level genome assembly based on ONT ultralong reads was performed using NextDenovo v2.5.0 (https://github.com/Nextomics/NextDenovo). Two assembly strategies were applied to the PacBio HiFi data: a HiFi‐only assembly and a hybrid HiFi + ONT assembly, both conducted using HiFiasm v0.18.9 (Cheng et al., 2021). The resulting contig‐level assemblies from all three strategies were evaluated using genome completeness BUSCO and contiguity metrics, and the highest quality assembly generated using the HiFi‐only strategy was selected as the T2T genomic backbone. To scaffold the diploid genome (2n), ALLHiC v0.9.8 (Zhang, Zhang, et al., 2019) was used to cluster the contigs into chromosomal groups using agglomerative hierarchical clustering. Within each n‐chromosome group, the contigs were ordered and oriented. Manual correction of the contig arrangement was performed using Juicebox v1.11.08 (Durand et al., 2016). Contigs with no Hi‐C contact signals but normal interactions with other sequences of the same size were considered likely heterozygous artifacts and were removed. Hi‐C interaction heat maps were generated using the 3D‐DNA visualization module (Dudchenko et al., 2017), ultimately anchoring a total of 962 044 720 bp contig sequences onto 12 chromosomes.

### Identification of telomeres and centromere regions

To identify telomeric regions, reads mapped to the chromosome termini were extracted, and the number of telomeric repeat motifs (‘CCCTAAA’/‘TTTAGGG’) in each read was quantified. The read with the highest motif count was designated as the reference telomeric read, whereas the other reads were treated as query reads. These reads were reassembled using Medaka_consensus v1.2.1 to generate consensus telomeric sequences. The resulting consensus sequences were then aligned to each chromosome using NUCmer v3.1 (Kurtz et al., 2004), and the best alignment results were used to replace the corresponding telomeric regions in the assembly to ensure accurate terminal reconstruction.

Homologs of Arabidopsis CENH3 (HTR12) were identified via BLASTP v2.6.0 (Camacho et al., 2009). Anti‐SxyCENH3 polyclonal antibodies were generated using the synthetic peptide (ARVKHFARRKLRGRQPS), corresponding to the 2nd to 18th amino acid residues of the predicted SxyCENH3 protein. The antibodies were custom synthesized and purified by AtaGenix Laboratories Co., Ltd. (Wuhan, PR China). ChIP assays were performed using SxyCENH3 antibodies. Young leaves of *S. xylocarpa* (10 g) were ground into a fine powder in liquid nitrogen. Chromatin was extracted with nuclear lysis buffer and fragmented using MNase (micrococcal nuclease). Fragmented chromatin was subjected to immunoprecipitation. ChIPed DNA and input DNA were submitted to Wuhan Frasergen Co., Ltd (Wuhan, PR China) for library construction and sequencing. IF was carried out following the protocol (Wang et al., 2023), with the following modification: seedlings were harvested when the primary roots reached approximately 0.5 cm in length, and the root tissues were fixed in 4% (v/v) formaldehyde for 10 min.

ChIP‐seq raw paired‐end reads and input reads were first processed using fastp v0.23.2 (Chen, 2025) to trim low‐quality bases and remove adapter contamination. The cleaned reads were then aligned to the genome using Bowtie2 v2.5.1 (Langmead & Salzberg, 2012). The resulting alignments were sorted and filtered to remove low‐quality mappings using Samtools v1.15.1 (Danecek et al., 2021). Genome‐wide enrichment profiles were generated using the bamCompare tool from the deepTools suite v3.5.1 (Ramírez et al., 2016) by calculating the log_2_ ratio of ChIP‐seq signal over input control. Putative centromeric regions on each chromosome were manually inspected and refined by examining signals and genomic features with the Integrative Genomics Viewer (IGV) v2.19.8 (Robinson et al., 2011), enabling precise delineation of centromere boundaries. The resulting data were visualized using pyGenomeTracks v3.9 (Lopez‐Delisle et al., 2021). The distribution of centromeric regions across chromosomes was visualized using RIdeogram (Hao et al., 2020).

### Repeat element identification, gene annotation, and non‐coding RNA annotation

Repeat elements were identified using a combination of de novo and structural approaches. RepeatModeler v2.0.4 (Flynn et al., 2020), LTR_FINDER (Xu & Wang, 2007), and LTRharvest v1.62 (Ellinghaus et al., 2008) were used to predict long‐terminal repeat (LTR) retrotransposons. Redundant LTR elements were filtered out using LTR_retriever v2.9.0 (Ou & Jiang, 2018). Subsequently, RepeatProteinMask v4.1.5 was used to mask the repeat sequences in the genome.

Gene structures were annotated by integrating evidence from three sources: transcriptome‐based predictions using TransDecoder v5.1.0, homology‐based alignments using Exonerate v2.4.0 (Slater & Birney, 2005), and *ab initio* gene predictions using augustus v3.3.2 and GlimmerHMM v3.0.4 (Delcher et al., 2007; Stanke et al., 2008). The completeness and accuracy of gene annotation were evaluated using BUSCO v5.2.2 (Manni et al., 2021).

Functional annotation of the predicted genes was carried out by sequence similarity searches against the Nr (Deng et al., 2006), Uniprot (Apweiler et al., 2004), and KEGG databases (Kanehisa & Goto, 2000), combined with conserved domain analysis using Pfam by InterProScan (Blum et al., 2021). Non‐coding RNAs, including tRNA genes, were identified using tRNAscan‐SE (Chan et al., 2021), whereas other non‐coding RNA species, such as snRNAs and miRNAs, were detected using INFERNAL v1.1.2 (Nawrocki & Eddy, 2013) by querying the Rfam database.

### Comparative genomics analysis

We selected 12 representative species from the Ericales to conduct comparative analysis with *S. xylocarpa*, including *Actinidia chinensis*, *A. rufa*, *Camellia lanceoleosa*, *C. sinensis*, *Davidia involucrata*, *Diospyros oleifera*, *Gilia yorkii*, *Primula veris*, *Rhododendron griersonianum*, *R. vialii*, *Vaccinium macrocarpon*, and *Vitellaria paradoxa*, together with three outgroups, *Amborella trichopoda*, *Oryza sativa*, and *Vitis vinifera*.

Orthologous gene families were identified using OrthoFinder (Emms & Kelly, 2019) based on the protein sequences of the 14 selected species (excluding the outgroup). BLASTP was used for sequence alignments. An ML phylogenetic tree was constructed using RAxML v8.2.10 based on a concatenated alignment of strictly single‐copy genes (genes present as single copies in approximately 90% of the species) (Stamatakis, 2014). MCMCTree from the PAML v4.9 package (Yang, 2007) was used to estimate species divergence times based on the ML tree. Gene family expansion and contraction events were inferred using CAFE v3.1 (Han et al., 2013).

We used minimap2 (Li, 2018) to align the two genomes of *S. xylocarpa* with the parameters “‐a ‐x asm5 ‐‐cs ‐r2k ‐‐eqx”, followed by identification of genomic structural variants using SyRi v1.6 (Goel et al., 2019).

### Collinearity, positively selected genes, and WGD detection

Protein sequences from different species were aligned using BLAST v2.6.0+ (Camacho et al., 2009), and genomic collinear blocks were identified using MCScanX (Wang et al., 2012). The synonymous substitution rate (*K*_s_), non‐synonymous substitution rate (*K*_a_), and their ratio (*K*_a_/*K*_s_) for collinear gene pairs were calculated using the yn00 module in PAML v4.9 (Yang, 2007).

Subsequently, gene sequence alignment between species was performed using LAST v1170 (Frith et al., 2010). Homologous gene pairs were mapped to genomic annotations using JCVI v0.9.13 (Tang et al., 2008) to determine their chromosomal positions and define collinear blocks based on adjacency.

A positive selection analysis was performed using CodeML in PAML v4.9 by applying likelihood ratio tests (LRTs) between the two models. Model A allowed ω > 1 on the foreground branches, whereas the null model restricted all sites to ω ≤ 1. Statistical significance was evaluated using the chi‐square distribution, and two genes were identified as being under significant positive selection (*P* < 0.05).

### Landscape of centromeres

Centromeric transposons were predicted and classified using the EDTA v2.27.0 pipeline (Ou et al., 2019). To further characterize these elements, LTR protein domains were identified using TEsorter in conjunction with the REXdb database (Neumann et al., 2019; Zhang et al., 2022). For enrichment signal statistics, Bedtools v2.31.1 (Quinlan, 2014) was used to generate 1‐kb windows, and multiBigwigSummary of deepTools v3.5.1 was employed to summarize enrichment values over these coordinates. Statistical analyses and graphical visualization were performed using R with the ggplot2 package.

### Population genetics analysis

Genomic variants were identified using GATK v4.5.0.0 (Mckenna et al., 2010) by applying hard‐filtering parameters as recommended by GATK. Annotated SNPs and small insertions/deletions (INDELs) were generated using ANNOVAR v20200607 (Wang et al., 2010). To ensure data quality, only biallelic SNPs with a minor allele frequency (MAF) ≥0.05 and a missing genotype rate ≤20% were retained using VCFtools v0.1.16 (Danecek et al., 2011) for the subsequent analyses.

The population structure was examined using a filtered SNP dataset. An ML phylogenetic tree was constructed using IQ‐TREE v2.3.6 (Minh et al., 2020), and PCA was performed using GCTA v1.93.2 (Yang et al., 2011). Genetic clustering analysis was conducted using ADMIXTURE v1.3.0 (Alexander et al., 2009) across *K* values from 2 to 9, with the optimal *K* determined by the lowest cross‐validation (CV) error.

Genome‐wide LD decay was calculated using PopLDdecay v3.41 (Zhang, Dong, et al., 2019), with pairwise *r^2^* values evaluated at 5, 10, and 300 kb. Selective sweep regions were identified by computing nucleotide diversity (π) and the fixation index (*F*_ST_) in sliding windows using VCFtools, retaining only those windows in which both π and *F*_ST_ values ranked within the top 10%. ROH were identified using PLINK v1.9 (Purcell et al., 2007). A sliding window of 50 SNPs was applied, allowing a maximum of one heterozygous site and five missing calls per window. The minimum length for an ROH was set to 100 kb, comprising at least 50 SNPs, and the maximum gap allowed for merging ROHs was 1000 kb. The total ROH length per individual was then divided by the total length of the genome (961 939 874 bp) to obtain the *F*_ROH_. Ho was calculated using the R package Gaston (https://cran.r‐project.org/web/packages/gaston). DSuite v0.5 r53 (Malinsky et al., 2021) and TreeMix v1.13 (Pickrell & Pritchard, 2012) were used to infer gene flow among the populations. Demographic history was reconstructed using the PSMC model (Li & Durbin, 2011), with a mutation rate of 4.58 × 10^−8^ per site per generation and a generation time of 10 years. Recombination rates were estimated in 100 kb windows across the genome using FastEPRR v2.0 (Gao et al., 2016). SIFT4G (Vaser et al., 2016) was used to predict deleterious mutations, and the deleterious mutation load in each 100 kb window was calculated as the ratio of deleterious sites to the number of coding sites within that window. IGV v2.19.8 (Robinson et al., 2011) was used to visualize the individual‐level genotypes of deleterious mutations across the population.

## AUTHOR CONTRIBUTIONS

Y‐GS, X‐LY, and N‐WL designed the research; AL, YJ, and Z‐WW collected the samples; S‐DL, S‐SZ, LW, and B‐JG performed the experiments and analyses; S‐SZ, LW, and N‐WL provided the funding; S‐DL, S‐SZ, LW, H‐HM, B‐JG, X‐LD, GK, N‐WL, X‐LY, and Y‐GS wrote the manuscript; and all authors contributed to its revision.

## CONFLICT OF INTEREST

The authors declare no competing interests.

## Supporting information


**Figure S1.** Length distribution of closely related species and *S. xylocarpa*.
**Figure S2.** Repeat sequence coverage per 5‐Mb window across 12 chromosomes.
**Figure S3.** Immunofluorescence assay of anti‐SxyCENH3 antibody on interphase of *S. xylocarpa*.
**Figure S4.** Genome‐wide distribution of SxyCENH3 enrichment in *S. xylocarpa*.
**Figure S5.** GO and KEGG enrichment analysis of different gene families.
**Figure S6.** LTR retrotransposon composition of SxyCENH3‐associated centromeric regions.
**Figure S7.** TE composition in the highly SxyCENH3 enriched region (log_2_(ChIP/Input) >1) on the right arm of chr1.
**Figure S8.** Annotation and feature of variation sites.
**Figure S9.** Variation distribution density.
**Figure S10.** Population genetic structure analysis based on ADMIXTURE.
**Figure S11.** Whole‐genome linkage disequilibrium (LD) analysis.
**Figure S12.** Selected regions in comparisons between NJ and different populations.
**Figure S13.** Determination of the optimal m value for TreeMix.
**Figure S14.** Genomic inbreeding estimates in *S. xylocarpa*.
**Figure S15.** The genomic comparison between two genomes of *S. xylocarpa*.
**Figure S16.** Genome−wide distribution of recombination rates (ρ).
**Figure S17.** Distribution of DNA repair genes harboring deleterious mutations across the 12 chromosomes of *S. xylocarpa*.
**Figure S18.** Deleterious mutation at position 54 236 363 within gene *SxylChr1G021390* (*POLH*) across all samples.

**Table S1.** Summary of the clean data sequenced by multiple technologies.
**Table S2.** Summary of assembly strategy‐based contig‐level genome assessment.
**Table S3.** Summary of chromosomes length and QV value.
**Table S4.** Summary of coding gene prediction results.
**Table S5.** Genes BUSCO evaluation results.
**Table S6.** Summary of the results of functional annotation of coding genes.
**Table S7.** Statistics on the results of non‐coding RNA annotation.
**Table S8.** Statistics on repeat sequence annotation.
**Table S9.** Statistics on telomere and centromere features.
**Table S10.** Summary of gene family clustering.
**Table S11.** The functional annotation of positive selection genes.
**Table S12.** Summary statistics of sampling and whole‐genome resequencing data per sample.
**Table S13.** Summary of variants of samples.
**Table S14.** Summary of variant functions.
**Table S15.** Summary of runs of homozygosity (ROH) for individuals of *S. xylocarpa*.
**Table S16.** Summary of nucleotide diversity (*π*) for populations of *S. xylocarpa*.
**Table S17.** Observed heterozygosity of each individual in chr1.
**Table S18.** Genomic proportions within peaks of the recombination rate distribution.
**Table S19.** The GO enrichment analysis of genes with deleterious mutations.

## Data Availability

The genome assembly has been deposited in the Genome Warehouse (GWH) at the National Genomics Data Center (NGDC) under PRJCA051182 (GWH accession: GWHJXXR00000000.1). The raw whole‐genome sequencing reads have been deposited in the Genome Sequence Archive (GSA) at NGDC under accession number CRA050003.
