# Supplementary material for: Telomere‐to‐telomere genome assembly of the endangered Jacktree (Sinojackia xylocarpa Hu) reveals its centromeric landscape and evolutionary dilemma as a small population
Source: Plant J. 2026 Nov 4;128(3):e71186. doi: 10.1111/tpj.71186 (PMC13732550; doi:10.1111/tpj.71186)
Supplement: Supplementary file 1 [file TPJ-128-0-s002.docx]

**SUPPORTING INFORMATION**

**Supplementary figures**

**Telomere-to-telomere genome assembly of the endangered Jacktree (*Sinojackia xylocarp*a Hu) reveals its centromeric landscape and evolutionary dilemma as a small population**

Si-Di Liang^1,2,#^, Si-Si Zheng^1,#^, Lei Wu^3,#^, Ang Liu^3^, Hong-Hu Meng^4^, Yun Jiang^1^, Zheng-Wei Wang^1^, Bin-Jie Ge^1^,Wen-Li Zou^5^, Qian Wan^5^, Xi-Ling Dai^2^, Gregor Kozlowski^1,6,7^, Nai-Wei Li^8,*^, Xiao-Ling Yan^1,*^, Yi-Gang Song^1,*^

^1^ Key Laboratory of National Forestry and Grassland Administration on East China Plant Conservation and Utilization, Shanghai Chenshan Botanical Garden, Shanghai 201602, China

^2^ College of Life Sciences, Shanghai Normal University, Shanghai 200234, China

^3^ College of Forestry, Central South University of Forestry and Technology, Changsha 410004, China

^4^ Plant Phylogenetics and Conservation Group, Centre for Integrative Conservation, Xishuangbanna Tropical Botanical Garden, Chinese Academy of Sciences, Kunming 650223, China

^5^ Administration Bureau of Longgan Lake National Nature Reserve, Hubei Province, Haungmei 43550, China

^6^ Department of Biology and Botanic Garden, University of Fribourg, Fribourg, Switzerland

^7^ Natural History Museum Fribourg, Fribourg, Switzerland

^8^ Institute of Botany, Jiangsu Province and Chinese Academy of Sciences (Nanjing Botanical Garden Mem. Sun Yat-Sen), Nanjing 210014, China

\# These authors contributed equally to this paper.

* For correspondence (e-mail: linaiwei8828@163.com (NWL), xlyan@cemps.ac.cn (XLY), and ygsong@cemps.ac.cn (YGS).

**Figure S1.** Length distribution of closely related species and *S. xylocarpa*.

**Figure S2.** Repeat sequence coverage per 5-Mb window across 12 chromosomes.

**Figure S3.** Immunofluorescence assay of anti-SxyCENH3 antibody on interphase of *S.xylocarpa*.

**Figure S4.** Genome-wide distribution of SxyCENH3 enrichment in *S. xylocarpa*.

**Figure S5.** GO and KEGG enrichment analysis of different gene families.

**Figure S6.** LTR retrotransposon composition of *SxyCENH3*-associated centromeric regions.

**Figure S7.** TE composition in the highly *SxyCENH3* enriched region (log_2_(ChIP/Input) > 1) on the right arm of chr1.

**Figure S8.** Annotation and feature of variation sites.

**Figure S9.** Variation distribution density.

**Figure S10.** Population genetic structure analysis based on ADMIXTURE.

**Figure S11.** Whole genome linkage disequilibrium (LD) analysis.

**Figure S12** Selected regions in comparisons between NJ and different populations.

**Figure S13.** Determination of the optimal m value for TreeMix.

**Figure S14.** Genomic inbreeding estimates in *S. xylocarpa*.

**Figure S15.** The genomic comparison between two genomes of *S. xylocarpa*.

**Figure S16.** Genome−wide distribution of recombination rates (ρ).

**Figure S17.** Distribution of DNA repair genes harboring deleterious mutations across the 12 chromosomes of *S. xylocarpa*.

**Figure S18.** Deleterious mutation at position 54,236,363 within gene SxylChr1G021390 (*POLH*) across all samples.

Figure S1. Length distribution of closely related species and *S. xylocarpa*. Distribution graphs for gene length, CDS length, intron length, and exon length of each species. The y-axes of the four graphs represent the percentage of genes within a given length category relative to the total number of genes. Different colored lines represent different species, with the red line indicating the species under study and other colored lines representing closely related species selected for annotation.

Figure S2. Repeat sequence coverage per 5 Mb window across 12 chromosomes.

Figure S3. Immunofluorescence assay of anti-SxyCENH3 antibody on interphase of *S.xylocarpa*. Chromosomes (blue) were stained with 4′,6-diamidino-2-phenylindole (DAPI). Scale bar, 10 μm.

Figure S4. Genome-wide distribution of SxyCENH3 enrichment in *S. xylocarpa*. Genome-wide distribution of SxyCENH3 occupancy across 12 chromosomes. The top track indicates annotated centromeric regions identified. The middle tracks show normalized read coverage from SxyCENH3 ChIP-seq and corresponding input DNA controls. The bottom track represents the log_2_-transformed ChIP/Input enrichment ratio, with positive values indicating SxyCENH3-enriched regions. Centromeric domains were defined based on broad and continuous *SxyCENH3* enrichment signals rather than isolated peaks.

Figure S5. (A) GO (left) and KEGG (right) enrichment results of unique gene families in comparison with all species. (B) GO (left) and KEGG (right) enrichment results of shared gene families in comparison with all species. (C) GO (left) and KEGG (right) enrichment results of significantly (p ≤ 0.05) expanded gene families. (D) GO (left) and KEGG (right) enrichment results of significantly (p≤0.05) contracted gene families. (E) GO (left) and KEGG (right) enrichment results of positively selected genes.

Figure S6. LTR retrotransposon composition of SxyCENH3-associated centromeric regions.

Figure S7. TE composition and abundance in the highly SxyCENH3 enriched region (log_2_(ChIP/ Input) > 1) on the right arm of chr1.

Figure S8. Annotation and feature of variation sites. (A) Annotation results of identified SNPs and INDELs (insertions, deletions) using ANNOVAR. (B) INDEL length distribution chart. (C) The proportion of various types of transversions and transitions in the overall variation.

Figure S9. Variation distribution density. Using a sliding window approach to divide the genome into 1Mb sub-regions, the number of SNPs (A), deletions (B), insertions (C), and indels (D) in each sub-region on the genome is counted separately, and a density map is drawn.

Figure S10. Population genetic structure analysis based on ADMIXTURE. (A) CV error values and maximum likelihood value distribution. (B) Genetic composition of each sample in the population under different K values.

Figure S11. Whole-genome linkage disequilibrium (LD) analysis. LD analysis using PopLDdecay, with *r*^2^ results at maximum distances of 10Kb (A), 30kb (B), 50kb (C), and 300Kb (D).

Figure S12. Selected regions in comparisons between NJ and different populations. Based on vcftools, calculate the *π* ratio and *F_ST_* value between each genome window of NJ and different populations. Select regions where both the π ratio and Fst value are in the top 10% as selected regions (above the blue line). (A) NJ vs GC. (B) NJ vs HM. (C) NJ vs NX. (D) NJ vs PZ. (E) NJ vs QY. (F) NJ vs XZ. (G) NJ vs YJ.

Figure S13. Determination of the optimal m value for TreeMix. (A) Mean likelihood and +/-SD for different m. (B) Determine the optimal m using OptM.

Figure S14. Genomic inbreeding estimates in *S. xylocarpa.* (A) Box plots of *F*_ROH_ across populations. (B) Mean and standard error (SE) of *F*_ROH_ across populations.

Figure S15. The genomic comparison between two genomes of *S. xylocarpa*. Whole-genome alignment between the two genomes was performed, and structural variations were detected using SyRI. The genome-wide distribution of syntenic regions and structural rearrangements, including inversions, translocations, duplications, and other rearrangements, is shown across the 12 chromosomes. Inversions are highlighted to indicate regions with reversed sequence orientation between the two genomes.

Figure S16. Genome-wide distribution of recombination rates (ρ). The red dashed line marks the 10th percentile (lowest decile), covering 10% of the genome. The bimodal distribution, with peaks at ~197 and ~387, suggests the presence of two distinct classes of recombination rate (ρ) within the genome.

Figure S17. Distribution of DNA repair genes harboring deleterious mutations across the 12 chromosomes of *S. xylocarpa*.

Figure S18. Deleterious mutation at position 54,236,363 within gene *SxylChr1G021390* (*POLH*) across all samples. At position 54,236,363, a C-to-G substitution is observed, which is predicted to be a deleterious mutation. Different colors denote alternative alleles (C/G), while the gray bar represents the reference genome sequence (C/C).
